# Stimulation of ROS Generation by Extract of *Warburgia ugandensis* Leading to G_0_/G_1_ Cell Cycle Arrest and Antiproliferation in A549 Cells

**DOI:** 10.3390/antiox10101559

**Published:** 2021-09-30

**Authors:** Yong-Li Zhang, Gui-Lin Chen, Ye Liu, Xiao-Cui Zhuang, Ming-Quan Guo

**Affiliations:** 1 Key Laboratory of Plant Germplasm Enhancement and Specialty Agriculture, Wuhan Botanical Garden, Chinese Academy of Sciences, Wuhan 430074, China; zhangyongli@wbgcas.cn (Y.-L.Z.); glchen@wbgcas.cn (G.-L.C.); liuye@wbgcas.cn (Y.L.); 2 The Sino-Africa Joint Research Center, Chinese Academy of Sciences, Wuhan 430074, China; 3 Innovation Academy for Drug Discovery and Development, Chinese Academy of Sciences, Shanghai 201203, China; 4 School of Chemical Biology and Environment, Yuxi Normal University, Yuxi 653100, China; zhuangxiaocui@yxnu.edu.cn

**Keywords:** *Warburgia ugandensis*, A549 cells, G_0_/G_1_ phase arrest, ROS, DNA damage

## Abstract

*Warburgia ugandensis* Sprague (WU) is a traditional medicinal plant used for the treatment of various diseases, including cancer, in Africa. This study aimed to evaluate the anti-non-small cell lung cancer (NSCLC) activities of WU against A549 cells and to reveal potential molecular mechanisms. The cytotoxicity of various WU extracts was evaluated with HeLa (cervical cancer), HepG2 (liver cancer), HT-29 (colorectal cancer), and A549 (non-small cell lung cancer) cells by means of Sulforhodamine B (SRB) assay. Therein, the dimethyl carbonate extract of WU (WUD) was tested with the most potent anti-proliferative activity against the four cancer cell lines, and its effects on cell viability, cell cycle progression, DNA damage, intracellular reactive oxygen species (ROS), and expression levels of G_0_/G_1_-related proteins in A549 cells were further examined. First, it was found that WUD inhibited the proliferation of A549 cells in a time- and dose-dependent manner. In addition, WUD induced G_0_/G_1_ phase arrest and modulated the expression of G_0_/G_1_ phase-associated proteins Cyclin D1, Cyclin E1, and P27 in A549 cells. Furthermore, WUD increased the protein abundance of P27 by inhibiting FOXO3A/SKP2 axis-mediated protein degradation and also significantly induced the γH2AX expression and intracellular ROS generation of A549 cells. It was also found that the inhibitory effect of WUD on the proliferation and G_0_/G_1_ cell cycle progression of A549 cells could be attenuated by NAC, a ROS scavenger. On the other hand, phytochemical analysis of WUD with UPLC-QTOF-MS/MS indicated 10 sesquiterpenoid compounds. In conclusion, WUD exhibited remarkable anti-proliferative effects on A549 cells by improving the intracellular ROS level and by subsequently modulating the cell proliferation and G_0_/G_1_ cell cycle progression of A549 cells. These findings proved the good therapeutic potential of WU for the treatment of NSCLC.

## 1. Introduction

*Warburgia ugandensis* Sprague (WU), also called pepper bark tree or greenheart tree, is a traditional and highly beneficial medicinal plant widely distributed in Eastern and Southern Africa. WU has been used to manage a broad range of diseases, including malaria, tapeworm, trypanosomiasis, leishmaniasis, bacterial infection, fungal infection, and cancer by traditional health practitioners in local African communities [1,2,3]. Presently, extensive studies of WU have focused on antibacterial activity and antifungal activity. However, only a few studies are associated with its anticancer activity, and some recent publications indicate that WU has been used for the treatment of breast cancer, cervical cancer, prostate cancer, skin cancer, throat cancer, intestinal cancer, and mouth epidermoid carcinoma, while the underlying molecular mechanisms remain unknown [1,4].

Lung cancer is one of the most common malignancies worldwide, with the new cases and deaths increasing year by year [5,6]. NSCLC is the most common form of lung cancer, accounting for approximately 85% of lung cancers [7]. In recent decades, there have been tremendous advances in the lung cancer therapy, but 75% of lung cancer patients are diagnosed at advanced stages, which means that the cancer cells have already spread to other parts of the body that are far away from the original tumor, so traditional chemotherapy still remains an effective strategy for treating lung cancer [8,9]. However, the efficacy of chemotherapeutic drugs is restricted due to acquired drug resistance and irreparable side effects [10]. Hence, it is urgent to develop new chemotherapeutic drugs for lung cancer treatment. Natural products are an important source of chemotherapeutic drugs, and about half of the anticancer drugs are either natural products or are directly derived from them [11]. The discovery of new chemotherapeutic drugs from natural products has attracted much attention because of their rich sources and enormous structural diversity.

Mammalian cell cycle progression and transitions are tightly orchestrated by cyclin-dependent kinases (CDKs), whose activity is modulated by several positive regulators (cyclins) and negative regulators, which are called CDK inhibitors (CDKIs). Uncontrolled cell cycle progression is a fundamental feature of NSCLC [12]. The G_1_/S transition is frequently dysregulated in NSCLC, which is associated with the upregulation of CDK activators (e.g., Cyclin D1 and Cyclin E1) and the downregulation of CDK inhibitors (e.g., P27 and P21) [13,14]. P27, a negative regulator of the G_1_/S transition, is frequently decreased in NSCLC, and the reduced expression of P27 in NSCLC correlates with tumor progression and poor prognosis [15,16]. The reduced P27 expression is mainly due to SKP2-mediated ubiquitin-proteasome degradation [17]. SKP2 (S-phase kinase-associated protein 2), a member of the F-box protein, has been reported to be up-regulated in NSCLC [18]. SKP2 functions as a critical component of the SCF E3 ligase complex, participating in the proteasomal degradation of P27 [19]. Apart from the post-translational regulation by SKP2, P27 is also regulated at the transcriptional level by FOXO3A, a principal transcription activator of P27 [20]. 

On the other hand, reactive oxygen species (ROS), a group of oxygen-containing ions and molecules with high reactivity, including superoxide (O_2_^−^, the precursor of most of the ROS), hydrogen peroxide (H_2_O_2_), the hydroxyl radical (•OH), and singlet oxygen (^1^O_2_), and they are essential for a variety of biological processes in normal and cancer cells [21,22]. Depending on the ROS level, ROS functions as a cancer-promoting or cancer-suppressing agent. At moderate concentrations, ROS activates the survival, angiogenesis, and metastasis of cancer cells, whereas high-level ROS-induced oxidative stress activates anti-tumorigenic signaling and induces cell senescence and cell death [21,23,24]. Cancer cells usually exhibit higher ROS level compared to normal cells, which makes cancer cells more vulnerable to further ROS insult. Therefore, inducing ROS generation can have a therapeutic effect on cancer cells.

Presently, this study intended to investigate the inhibitory ROS-dependent growth and cell cycle arrest effects of WUD on A549 cells and managed to reveal the molecular mechanisms.

## 2. Materials and Methods

### 2.1. Chemicals and Reagents

Ham’s F-12K medium, McCoy’s 5A medium, Dulbecco’s modified Eagle’s medium (DMEM), Minimum Essential Medium (MEM), 100 U/mL streptomycin, and 100 μg/mL penicillin were acquired from Gibco (Life Technologies, Waltham, MA, USA). Fetal bovine serum was obtained from BI (Biological Industries, Beit Haemek, Israel). An SRB assay kit was purchased from Bestbio (Shanghai, China). Cell cycle and apoptosis analysis kits were purchased from Yeasen Biotech (Shanghai, China). A Reactive Oxygen Species Assay Kit, cycloheximide (CHX), and Hoechst 33258 were obtained from Beyotime (Beyotime Biotechnology, Shanghai, China). Normal goat serum was purchased from Boster (Boster Biological Technology, Wuhan, China). Anti-Cyclin D1, anti-Cyclin E1, anti-P27, anti-GAPDH, anti-β-actin, anti-FOXO3A, anti-SKP2, anti-γH2AX, HRP-conjugated affinipure goat anti-rabbit, HRP-conjugated affinipure goat anti-mouse, and CoraLite488-conjugated affinipure goat anti-rabbit were purchased from Protein Tech (Proteintech group, Wuhan, China). Ultrapure water was produced with an ultrapure water system (Yipu Yida Technology, Nanjing, China). All of the analytically pure chemicals and solvents were purchased from Sinopharm Group (Shanghai, China).

### 2.2. Plant Material

The WU root bark was collected in August 2015 from Mountain Kenya, Kenya. Prof. Guangwan Hu, a senior taxonomist of the Wuhan Botanical Garden, Chinese Academy of Sciences, authenticated the plant, and a voucher specimen (No. 2015001) was deposited in the herbarium of the Key Laboratory of Plant Germplasm Enhancement and Specialty Agriculture of the Chinese Academy of Sciences.

### 2.3. Sample Preparation and Phytochemical Analysis

Air dried WU root bark (50.0 g) was ground into a fine powder and was ultrasonically extracted for 0.5 h (three times) at room temperature with dimethyl carbonate, ethanol, and water, respectively. The obtained extract solution was filtered and evaporated under vacuum to afford the crude extracts WUD, WUEt, and WUH. The WUD samples were further subjected to UPLC-MS analysis by coupling the Agilent 1290 Infinity II system to the Q-TOF Premier mass spectrometer G6530C (Agilent Technologies Inc., Santa Clara, CA, USA) with electrospray ionization interface (ESI) in the negative ionization mode, and the UPLC conditions were set below this value using an ACQUITY UPLC BEH column (50 mm × 2.1 mm, 1.7 µm, ACQ, Waters Co., Milford, MA, USA): the column temperature was set to 30 °C; the gradient elution consisted of 0.1% formic acid in water (A) and acetonitrile (B) with a gradient as follows: 0–20 min, 20% B; 20–25 min, 20–30% B; and 25–45 min 30–32.6% B, with a flow rate of 0.2 mL/min.

### 2.4. Cell Line and Cell Culture

HeLa (human cervical carcinoma), HepG2 (human hepatocellular carcinoma), HT-29 (human colorectal adenocarcinoma), and A549 (human non-small cell lung carcinoma) cell lines were maintained with MEM medium, DMEM medium, McCoy’s 5A medium, and Ham’s F-12K medium, respectively. All of the cell lines were cultured in medium supplemented with 10% fetal bovine serum and streptomycin/penicillin at 37 °C in an incubator with a 5% CO_2_ atmosphere.

### 2.5. Cell Viability Assay

Cell viability was quantified using Sulforhodamine B (SRB) (Bestbio, Shanghai, China) assay. The WU extracts WUD, WUEt, and WUH were dissolved in DMSO (100 mg/mL) and were then diluted in the cell culture media (MEM medium for HeLa, DMEM medium for HepG2, McCoy’s 5A medium for HT-29, and Ham’s F-12K medium for A549) before the cells were added. The final concentration of DMSO in the cells was less than or equal to 0.1% (*v*/*v*). Briefly, 1.0 × 10^4^ cells in 100 μL culture medium were seeded into 96-well plates and were incubated with individual treatment. After incubation, cell viability was detected following the manufacturer’s protocols.

### 2.6. Cell Cycle Analysis

The cell cycle was analyzed using a cell cycle and apoptosis kit (Yeasen Biotech, Shanghai, China) by flow cytometry (BD Accuri C6, San Jose, CA, USA). Briefly, A549 cells were seeded in 6 well tissue culture plates and were incubated overnight. Following treatment, the cells were collected, washed with ice-cold PBS buffer, and fixed with 70% ethanol at −20 °C overnight. After fixation, the cells were stained with propidium iodide (PI) in PBS containing RNase A for 30 min. The DNA content of cells was analyzed by flow cytometry.

### 2.7. Western Blotting

A549 cells were washed with pre-cold PBS and lysed with RIPA lysis buffer (Beyotime Biotechnology, Shanghai, China), and the protein concentration was determined with a BCA protein assay kit (Beyotime Biotechnology, Shanghai, China). Samples were separated by means of electrophoresis in SDS-PAGE and were then transferred to nitrocellulose membranes and were incubated with the indicated primary antibodies and horseradish peroxidase (HRP)-conjugated secondary antibodies. The specific protein bands were detected with the ECL chemiluminescence system (Merck Millipore, Darmstadt, Germany).

### 2.8. Immunofluorescence Assay

A549 cells grown on coverslips were incubated with varying concentrations of WUD for 24 h. Cells were washed with PBS, fixed with 3.7% paraformaldehyde for 10 min, and then permeabilized with 0.1% Triton X-100 for 5 min followed by blocking with 1% normal goat serum for 60 min at 37 °C. Afterward, cells were incubated with the primary antibody anti-γH2AX at 37 °C for 2 h and then incubated with the secondary antibody at 37 °C for 1 h. The nuclear DNA was stained with Hoechst 33258 at 37 °C for 5 min. Images were captured using a Leica TCS SP8 confocal microscope (Leica, Wetzlar, Germany).

### 2.9. Measurement of ROS Generation

ROS determination was performed by using a reactive oxygen species (ROS) assay kit (Beyotime, Shanghai, China) according to the manufacturer instructions. Briefly, cells were seeded into 96 well plates (2.0 × 10^4^ per well) and were incubated overnight for adhesion. Then, the cells were treated with varying concentrations of WUD for 24 h. Afterward, the cells were incubated with DCFH-DA (10 µM) for 20 min in the dark. The DCF fluorescent signals were analyzed by a Tecan microplate reader (Tecan Infinite M200 Pro, Tecan Group Ltd., Männedorf, Switzerland) and a cell imaging multi-mode reader (Cytation 1, BioTek, Winooski, VT, USA).

### 2.10. Statistical Analysis

Data are expressed as mean ± standard deviation (SD). Statistical analyses were performed using an unpaired two-tailed Student *t*-test. *p*-values < 0.05 were considered statistically significant.

## 3. Results and Discussion

### 3.1. Effect of WU Extracts on the Growth of Human Cancer Cell Lines

**Figure 1:**
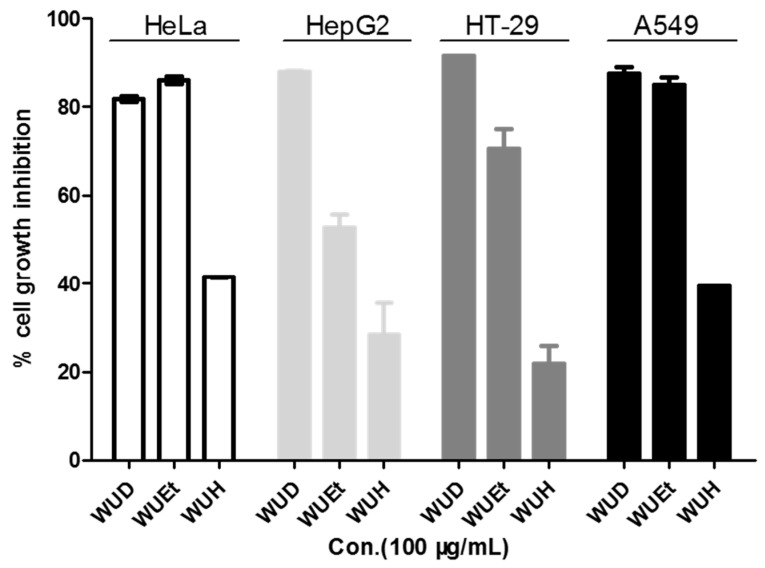
Inhibitory growth effects of three different WU extracts against four different cancer cell lines. HeLa, HepG2, HT-29, and A549 cells were treated with 100 µg/mL WU extracts (WUD, WUEt, and WUH) for 48 h. Cell viability was determined by SRB assay.

WU is widely used by local medicine practitioners in Africa to treat various diseases, including cancer. To investigate the anti-cancer potentials of WU, the WU extracts WUD, WUEt, and WUH were tested for inhibitory cell growth activity on HeLa, HepG2, HT-29, and A549 cells. As shown in Figure 1, WUH showed the least cytotoxic effect among the three different WU extracts on all four cell lines at the concentration of 100 μg/mL, while WUD exhibited the most potent inhibitory growth activity against the cancer cells, with a cell growth inhibition rate exceeding 80% against all four cell lines. As for WUEt, the HeLa and A549 cells were more sensitive than HepG2 and HT-29, with cell growth inhibition rates of 85.93 ± 0.70%, 85.05 ± 1.34%, 52.72 ± 2.10%, and 70.60 ± 3.67%, respectively. In this context, the most potent WUD extracted was selected for further study against A549 cells.

Up until now, there have been about 90 compounds isolated from WU, which are mainly composed of sesquiterpenes, flavonoids, fatty acids, lignanamides, and essential oils [25,26,27,28,29]. Some of the compounds isolated from other medicinal plants exhibiting anticancer activity have also been found in WU, such as cinnamosmolide, 6β-acetoxyisodrimenin, ungandensolide, futronolide, warburganal, polygodial, mukaadial, drimenol, and cinnamodial [30,31,32,33,34,35,36].

WU is a valuable medicinal plant that can be used to manage a wide range of health issues. As all parts of WU taste hot and peppery, the leaves, bark, young shoots, and fruits of WU are used to add flavor to curries, whereas the roots are usually used in soup, suggesting that WU is safe for humans.

The most commonly used medicinal parts of WU are the bark, roots, and leaves of the plant, of which the bark is the most commonly used part [2,28]. Garbe et al. conducted a broader ethnobotanical survey of 16 medicinal plants that are frequently used for the treatment of a variety of medical disorders, including cancer. In this report, WU was used to manage a broad range of cancers, but the researchers did not use the same parts or preparing methods [1]. Choi et al. reported that the methanol extract of WU stem bark exhibited potent anti-proliferation activity against human hepatocellular carcinoma HepG2 cells with a IC_50_ value of 20.6 ± 0.2 μg/mL [37]. Xu et al. also reported the strong cytotoxicity activity of WU bark extract against mouth epidermoid carcinoma KB cells [33]. As shown in Figure S1, the anti-proliferative effects of stem bark extracts and root bark extracts of WU against A549 cells were evaluated by SRB assay, and the results indicated that root bark extracts exhibited more potent anti-proliferative activity compared to stem bark extracts.

In Africa, the bark, roots, and leaves of WU are used to treat many ailments. Due to the unsustainable harvesting of the bark and roots along with the fact that the natural propagation of WU is problematic, its availability has been declining throughout its natural habitats [38,39]. In this regard, a researcher from the Wuhan Botanical Garden, Chinese Academy of Sciences, established an efficient protocol for the regeneration of WU through tissue culture, which will facilitate the recovery of WU and will also provide an alternative method for obtaining WU for phytochemical research and medicinal study [40].

### 3.2. WUD Reduces A549 Cell Viability in a Dose- and Time-Dependent Manner

**Figure 2:**
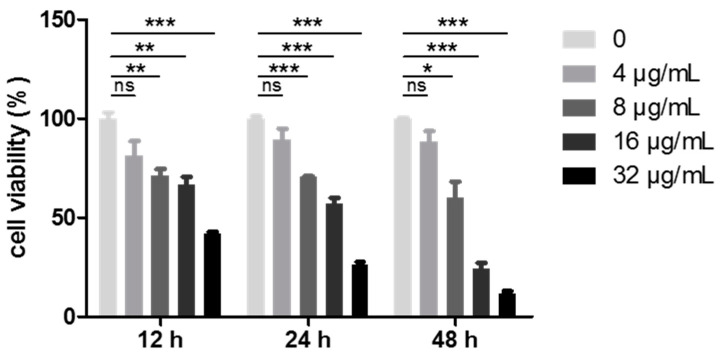
WUD inhibited A549 proliferation. A549 cells were treated with 0, 4, 8, 16, and 32 μg/mL WUD. Cell viability was determined by SRB assay at the indicated time points. * *p* < 0.05; ** *p* < 0.01; *** *p* < 0.001. ns: not significant.

Given the most potent cell growth inhibition rates of the WUD among the four different human cancer cell lines and the fact that lung cancer remains one of the most malignant tumors with an extremely high rate of morbidity and mortality, the cytotoxicity of WUD against A549 was then further tested. As presented in Table 1, WUD was the most potent extract, with an IC_50_ value of 7.13 ± 2.40 µg/mL, followed by WUEt, with an IC_50_ value of 32.28 ± 9.13 µg/mL. In order to evaluate the kinetics of WUD-induced A549 anti-proliferation, A549 cells were treated with various concentrations of WUD at the indicated time points, and the cell viability was measured via SRB assay. As shown in Figure 2, WUD depressed the cell viability of A549 cells in a dose- and time-dependent manner. In this regard, the remarkable anti-proliferative activity of WUD against A549 cells partly explains its good potential as an empirical natural medicine for the treatment of cancers in African communities.

Medicinal plants are important sources of chemotherapeutic drugs. In addition to WU, some plant extracts inhibit the proliferation of NSCLC cell lines (see below Table S1). According to Table S1, 26 extracts of 16 plants are reported to exhibit anti-NSCLC activity. Eleven of these extracts from seven plants display IC_50_ values below 100 µg/mL: *Fritillariae cirrhosae*, *Elephantopus mollis*, *Eucalyptus globulus*, *Bupleurum scrozonerifolium*, *Selaginella doederleinii*, and *Alnus nitida*, *Punica granatum.* Among them, the methanol extract from *Alnus nitida* leaves and stem bark shows the most potent inhibitory growth activity against NSCLC A549 cells and H460 cells, with IC_50_ values ranging from 10.67 ± 0.12 μg/mL (against H460, 48 h) to 25.47 ± 0.13 µg/mL (against H460, 48 h). The IC_50_ value of WUD against A549 cells is 7.13 ± 2.40 µg/mL, which is lower than that of *Alnus nitida*, suggesting the potential of WU for the treatment of NSCLC.

### 3.3. WUD Induces G_0_/G_1_ Phase Arrest in A549 Cells

**Figure 3:**
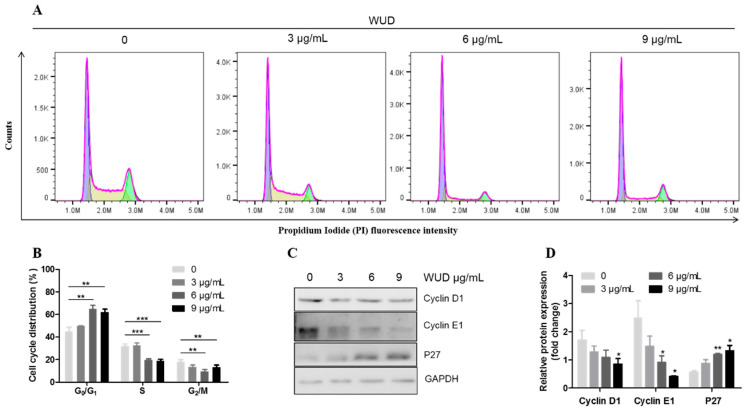
WUD induced cell cycle arrest at the G_0_/G_1_ phase of A549 cells. (**A**,**B**) A549 cells were treated with 0, 3, 6, and 9 μg/mL WUD for 24 h, and the DNA content was analyzed using flow cytometry. (**C**,**D**) The expression of G_0_/G_1_ phase-related proteins was determined by Western blot. * *p* < 0.05; ** *p* < 0.01; *** *p* < 0.001.

In order to investigate the mechanism underlying the WUD-induced cell growth inhibition of A549, the effect of WUD on cell cycle distribution was analyzed using flow cytometry. As depicted in Figure 3A,B, treatment with WUD for 24 h led to cell cycle arrest at the G_0_/G_1_ phase with a concomitant decrease in the S phase and G_2_/M phase compared to untreated cells. The cell populations of the G_0_/G_1_ phase increased from 44.73 ± 3.07% in the control cells and to 61.67 ± 2.44% in the A549 cells treated with 9 µg/mL WUD for 24 h, whereas the cell populations of the S phase and G_2_/M phase decreased from 31.77 ± 1.53% to 18.80 ± 1.13% and 18.23 ± 1.39% to 13.00 ± 1.68%, respectively (Figure 3A,B). To further explore WUD-induced G_0_/G_1_ phase cell cycle arrest, we examined the protein expression level of G_0_/G_1_ phase-related proteins, including Cyclin D1, Cyclin E1, and P27. WUD treatment significantly decreased the Cyclin D1 and Cyclin E1 protein expression but increased P27 expression, wherein 9 µg/mL WUD reduced the expression of Cyclin D1 and Cyclin E1 by 0.51 fold and 0.17 fold, respectively, while increasing the expression of P27 by 2.32 fold compared to untreated cells (Figure 3C,D). Collectively, these data revealed that WUD induced G_0_/G_1_ cell cycle arrest and modulated the protein expression of Cyclin D1, Cyclin E1, and P27 in A549 cells. This implies that the treatment of A549 cells with WUD could interfere with the synthesis of DNA and could inhibit A549 cell growth at this stage. 

### 3.4. WUD Enhances the Stability of P27 in A549 Cells

**Figure 4:**
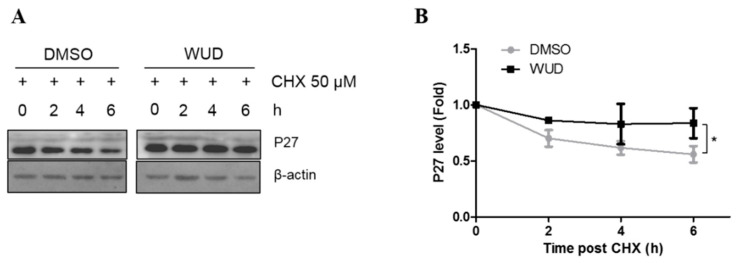
WUD-induced P27 accumulation was associated with protein degradation inhibition. (**A**,**B**) A549 cells were incubated with WUD (0, 9 µg/mL) followed by a combination treatment of WUD and CHX (50 µM) for the indicated intervals. A protein level of P27 was determined by Western blot. * *p* < 0.05.

P27 is a cyclin-dependent kinase inhibitor and plays an important role in multiple cellular processes, including cell cycle progression, apoptosis, autophagy, cell senescence, cell differentiation, migration, and invasion [41,42,43,44]. P27 is frequently decreased in NSCLC, and the reduced expression of P27 correlates with tumor progression and poor prognosis [15,16]. Since the above results demonstrated that WUD induced cell cycle arrest in the G_0_/G_1_ phase, which was concomitant with an increase in P27, the A549 cells were treated with CHX, a protein synthesis inhibitor for the indicated intervals so as to determine the mechanism by which WUD treatment increased the protein expression of P27. As shown in Figure 4, compared to the control cells, the protein expression of P27 was elevated (Figure 4A), and the P27 decline was significantly blocked in the WUD-treated cells (Figure 4B).This clearly indicates that WUD induced P27 accumulation by enhancing the stability of P27 in the A549 cells. 

### 3.5. FOXO3A/SKP2 Axis Involves in WUD-Induced P27 Accumulation

**Figure 5:**
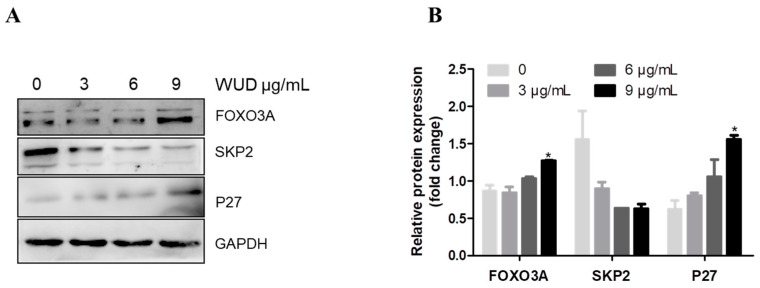
Effect of WUD treatment on FOXO3A, SKP2, and P27 expression. (**A**,**B**) A549 were treated with 0, 3, 6, and 9 μg/mL WUD for 24 h. The protein expression of FOXO3A, SKP2, and P27 was determined by Western blot. * *p* < 0.05.

For the further underlying mechanism of WUD-induced P27 accumulation, some key factors involved in the regulation of P27 accumulation were then evaluated. FOXO3A is a negative regulator of SKP2, which controls the ubiquitylation and degradation of P27. To determine the underlying mechanism of WUD-induced P27 accumulation, Western blot assay was performed to detect the protein expression of SKP2 and FOXO3A upon WUD treatment. As displayed in Figure 5, increasing the dose of WUD significantly increased the protein expression of FOXO3A and P27, suppressed the expression of SKP2 (Figure 5A), wherein 9 µg/mL WUD reduced the expression of SKP2 by 0.41 fold, and increased the expression of FOXO3A (1.48 fold) and P27 (2.51 fold) compared to untreated cells (Figure 5B). These results indicate that the FOXO3A/SKP2 axis was implicated in the up-regulation of P27 induced by WUD treatment at a relatively high concentration (9 µg/mL), which induced the consequent G_0_/G_1_ phase arrest in A549 cells.

P27 abundance is regulated by transcriptional and post-translational mechanisms [17]. Transcription factors such as Myc, FOXO, and Menin are implicated in the transcriptional regulation of P27. Myc, a protooncogene, downregulates P27 via reducing P27 transcription, increasing microRNAs that target P27, and facilitating P27 degradation [17,45,46,47,48,49,50] while FOXO and Menin are both positive regulators of P27 that increase the P27 transcription level [51]. Therefore, in addition to regulation at the post-translational level, WU might also affect the transcription of P27 in A549 cells.

### 3.6. WUD Induces DNA Damage in A549 Cells

**Figure 6:**
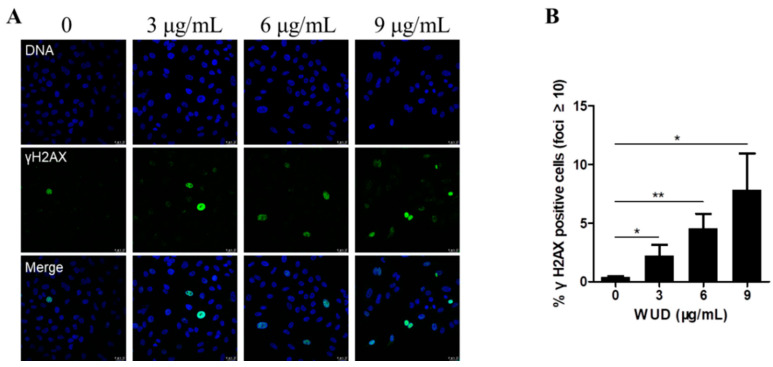
WUD induced DNA damage response in A549 cells. (**A**) Representative images of A549 cells with γH2AX foci after exposure to WUD (0, 3, 9 µg/mL). (**B**) γH2AX positive cells (≥10 foci per nucleus) were quantified and graphed. * *p* < 0.05; ** *p* < 0.01.

DNA damage caused by multiple environmental and intrinsic sources results in various cellular responses, including cell cycle arrest, cellular senescence, and different cell death programs [52]. Since WUD could induce the G_0_/G_1_ cell cycle arrest of A549 cells, we decided to investigate the effect of WUD on the DNA damage of A549 cells. As displayed in Figure 6, after treatment with WUD, the A549 cells showed a dramatic dose-dependent increase in the expression of γH2AX (a marker of double stranded DNA damage) (Figure 6A), as shown by 18.73-fold upregulation of the γH2AX positive cells after incubation with 9 µg/mL WUD compared to the untreated cells (Figure 6B). This clearly indicates that WUD could induce the DNA damage response in A549 cells.

### 3.7. WUD Stimulates Cellular ROS Generation in A549 Cells

**Figure 7:**
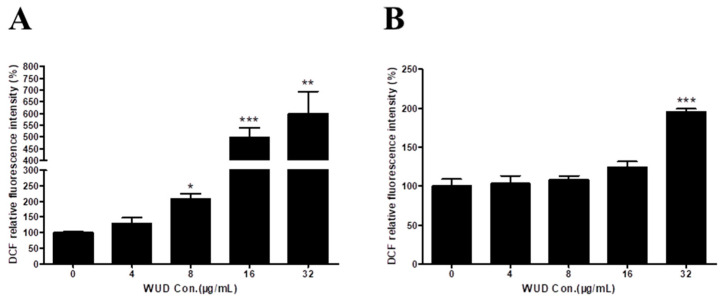
WUD stimulated the intracellular ROS generation of A549 cells. A549 cells were pretreated with DMSO or NAC (5 mM) for 1 h before being co-treated with WUD (0, 4, 8, 16, and 32 µg/ mL) for 24 h. ROS level in A549 cells was determined by DCF without (**A**) or with NAC pretreatment (**B**). * *p* < 0.05; ** *p* < 0.01; *** *p* < 0.001.

On the other hand, elevated ROS levels may lead to oxidative DNA damage. Given that WUD induced DNA damage in A549 cells, we decided to detect the effect of WUD on ROS level in A549 cells. The ROS level was determined by DCFH-DA staining via a fluorescence microplate reader. As revealed in Figure 7, compared to the control cells, the intracellular ROS was significantly increased in the WUD-treated cells in a concentration-dependent manner (Figure 7A) and could be reversed by the addition of NAC in a relatively low concentration (4, 8, and 16 µg/mL) of WUD-treated cells (Figure 7B). 

Compared to normal cells, cancer cells usually contain a higher basal level of ROS, which makes cancer cells are more vulnerable to damage by further ROS insults. Therefore, the manipulation of the ROS levels is a promising strategy for cancer therapy [53]. Natural products derived from medicinal plants have been used for anticancer therapy based on ROS-induced cell death [54,55,56]. Stimulating ROS generation is also a widely accepted mechanism that is manipulated by a variety of anti-NSCLC chemotherapeutical drugs, including cisplatin, etoposide, doxorubicin, docetaxel, and paclitaxel [57,58,59,60,61,62].

To avoid high levels of ROS-induced cell death, cancer cells maintain a highly activated antioxidant defense system [63]. Nuclear factor erythroid 2-related factor 2 (NRF2), the master regulator of the antioxidant defense system, targets genes that contribute to the detoxification of intracellular ROS [64]. Under unstressed conditions, NRF2 is sequestered in the cytoplasm by its main repressor Keap1 and is then subjected to ubiquitylation and proteasomal degradation, thus maintain a relatively low expression level [65]. In response to a variety of stresses, including excessive induced-oxidative stress, NRF2 dissociates with Keap1 and then translocates into the nucleus, where NRF2 dimerizes with small Maf proteins and activates target gene expression [65]. Besides Keap1, GSK3β also negatively regulates NRF2 via directly phosphorylating the Neh6 degron of NRF2 and the promoting ubiquitin E3 ligase β-TrCP recognizes NRF2 and mediates its degradation [64]. It is well known that GSK3β is negatively regulated by PI3K/Akt signaling, a vital pro-survival signaling pathway that is frequently activated in human cancers, including NSCLC [64,66]. In addition to positively regulating NRF2, Akt is also a negative regulator of P27 and a positive regulator of Cyclin D1 [67,68,69,70]. This might partly explain how WUD decreased Cyclin D1 expression while increasing P27 abundance. Therefore, further studies are still needed to verify whether the anti-NSCLC activity induced by WUD is related to PI3K/Akt signaling or other signaling pathways.

Apoptosis is a well-characterized type of programmed cell death (PCD) that includes mitochondrial-dependent apoptosis (intrinsic apoptosis) and death receptor-dependent apoptosis (extrinsic apoptosis). The intrinsic apoptosis pathway is governed by the pro-apoptotic (BAX and BAK) and the anti-apoptotic proteins (Bcl-2 and Bcl-xL) of the Bcl-2 family. The Bcl-2 family proteins regulate mitochondrial membrane permeability (MMP) according to the ratio of pro-apoptotic and anti-apoptotic proteins. The activation of BAK and BAX leads to mitochondrial outer membrane permeabilization (MOMP), which leads to cytochrome c release from the mitochondria to the cytoplasm and the activation of caspase 9 and caspase 3 [71]. Intracellular ROS are mainly produced from the mitochondria and plays a key role in intrinsic apoptosis execution. Extrinsic apoptosis is regulated through the tumor necrosis factor α (TNFα) pathway and the Fas pathway. ROS was shown to facilitate extrinsic apoptosis by activating TNFα signaling [72]. As WUD increases the intracellular ROS level, it is necessary to detect whether WUD leads to the mitochondria dysfunction, mitochondrial-dependent apoptosis, and extrinsic apoptosis. To investigate whether the anti-proliferative effect induced by WUD was related to apoptosis, an Annexin V-FITC/PI double staining followed by flow cytometry was conducted. As shown in Figure S2, no significant differences in the percentage of apoptotic cells were found between the control cells and the WUD-treated cells. The results suggest that WUD treatment may not activate apoptosis in A549 cells.

Autophagy is a self-degradative process that plays a housekeeping role by removing unnecessary or dysfunctional components through a lysosome-dependent regulated mechanism to facilitate cell survival. For cancer cells, autophagy is not only considered to be a cell survival mechanism but also a tumor suppressor mechanism that induces cell death. It was reported that ROS involves in autophagy via decreasing the activity of the mammalian target of rapamycin (mTOR), the central negative regulator of autophagy, or by targeting Beclin-1 (an important autophagy-related protein) and ATG4 (another autophagy-related protein) [21]. Therefore, further studies are still needed to verify whether the anti-NSCLC activity induced by WUD is related to autophagy.

### 3.8. Involvement of ROS in WUD-Modulated Cell Proliferation and Cell Cycle Progression of A549 Cells

**Figure 8:**
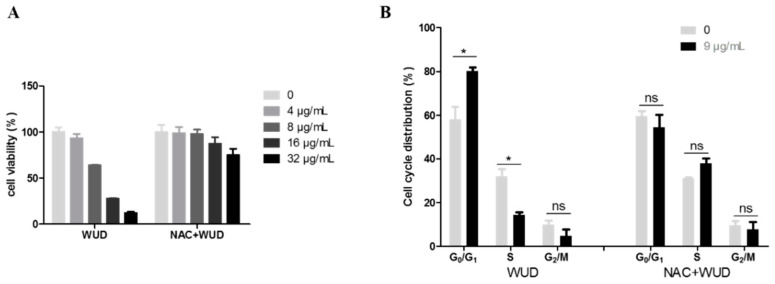
ROS was involved in the regulation of cell proliferation and cell cycle progression of A549 cells caused by WUD. A549 cells were pretreated with DMSO or NAC (5 mM) for 1 h before co-treated with WUD for 48 h. (**A**) Cell viability was assessed SRB assay. (**B**) The cell cycle was analyzed using flow cytometry. * *p* < 0.05. ns: Not significant.

To investigate whether ROS is implicated in WUD-induced anti-proliferation and cell cycle arrest, cell viability and cell cycle progression were characterized by means of SRB assay and flow cytometry, respectively. The concentration-dependent antiproliferative effect caused by WUD treatment could be significantly mitigated by NAC treatment (Figure 8A). The treatment of A549 cells with 9 µg/mL WUD for 48 h increased the number of cells in the G_0_/G_1_ phase remarkably and simultaneously decreased the number of cells in the S phase (Figure 8B). Similarly, the WUD-induced cell cycle arrest was counteracted by pretreatment with NAC (Figure 8B). These results indicate that ROS was involved in the WUD-modulated cell proliferation and cell cycle progression of A549 cells.

Besides WU, *Brucea javanica* oil (BJO) and luteoloside derived from *Gentiaana macrophylla* could also increase the intracellular ROS level and the increased ROS, leading to mitochondria-mediated apoptosis or PI3K/Akt/mTOR/p70S6K-modulated autophagy. *Brucea javanica* oil (BJO), a traditional herbal medicine extracted from *Brucea javanica* seeds, has been clinically used in combination with chemotherapy or radiotherapy to treat NSCLC [73]. BJO induces G_0_/G_1_ arrest and apoptosis via ROS-mediated mitochondrial dysfunction in NSCLC A549 cells and small cell lung cancer (SCLC) H446 cells. The BJO-partly induced G_0_/G_1_ arrest via the modulation of P53 and Cyclin D1, which was different from the WUD-modulated Cyclin D1, Cyclin E1, and P27 expression. Luteoloside, a flavonoid derived from the medicinal plant *Gentiaana macrophylla* has been reported to induce NSCLC cell death via inducing autophagy, G_0_/G_1_ arrest, and intracellular ROS generation [74]. Moreover, the luteoloside-induced ROS functions as an upstream suppressor of the PI3K/AKT/mTOR/p70S6K pathway, which is involved in luteoloside-induced autophagy. Luteoloside-induced G_0_/G_1_ arrest is accompanied by the reduced expression of CyclinE, CyclinD1, and CDK4.

### 3.9. Identification of Main Components in WUD by UPLC-QTOF-MS/MS

**Figure 9:**
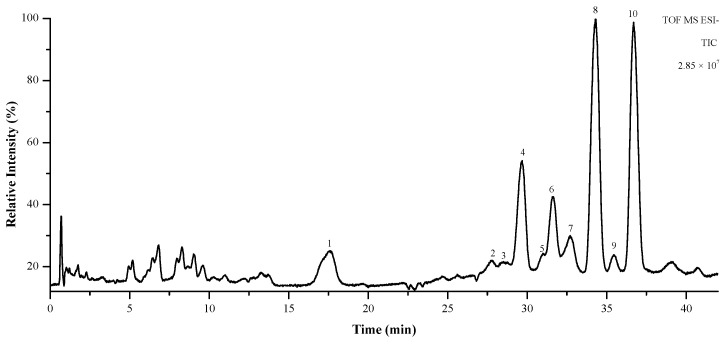
Total ion chromatogram (TIC) of WUD in the negative ion mode.

To further identify and characterize the phytochemical constituents in WUD that are potentially responsible for the activities stated above, an analysis of the phytochemicals in WUD was conducted with UPLC-QTOF-MS/MS. As shown in Figure 9, a total of 10 peaks were detected and then tentatively identified, as shown in Table 2. These 10 compounds belong to sesquiterpenoids, which are rich in WU and might be its main anti-proliferative components [75].

Up until now, extensive studies on WU have focused on antibacterial and antifungal activities, but only a few studies have considered its anticancer activity. In addition, some recent publications have reported that the WU stem and bark extracts exhibit potent anti-proliferative activity against HepG2 and KB cells, respectively [33,37]. However, those studies did not uncover the underlying molecular mechanisms of its action. This work demonstrated that root bark extracts exhibit more potent anti-proliferative activity compared to stem bark extracts (Figure S1), and this is the first report that has demonstrated that WUD exhibits remarkable anti-proliferative effects on A549 cells by improving the intracellular ROS level and by subsequently modulating the cell proliferation and G_0_/G_1_ cell cycle progression of A549 cells.

## 4. Conclusions

WU has been long used as an important traditional herbal medicine for the treatment of various diseases, including cancer, in Africa. The study aimed to evaluate the anti-NSCLC activity of WU against A549 cells and to reveal a potential molecular mechanism. In this work, WUD was first revealed to exhibit remarkable anti-proliferative effects on A549 cells by improving the intracellular ROS level and by subsequently modulating the cell proliferation and G_0_/G_1_ cell cycle progression of A549 cells. Furthermore, the FOXO3A/SKP2 axis was implicated in the up-regulation of P27 upon treatment with WUD at a relatively high concentration (9 μg/mL). Collectively, these findings imply the good therapeutic potential of WU that should be further developed for the treatment of NSCLC.

## Figures and Tables

**Table 1:** Cytotoxicity of WU extracts toward A549 cell lines determined by SRB assay after 48 h incubation.

Fraction	WUD	WUEt	WUH
IC_50_ (µg/mL).	7.13 ± 2.40	32.28 ± 9.13	>100

**Table 2:** Analysis of WUD with UPLC-QTOF-MS/MS.

No.	Rt. (min)	Formula	Theoretical [M–H]^−^	Experimental [M–H]^−^	Error (ppm)	Fragment Ions (MS/MS)	Identification
1	17.870	C_15_H_22_O_4_	265.1445	265.1443	−0.8	265.1438, 221.1551, 205.1239, 191.1432, 177.1282, 161.0963, 147.1230, 106.0388	Dendocarbin L
2	27.275	C_15_H_20_O_3_	247.1340	247.1342	0.8	247.1344, 229.1210, 214.0999, 205.1602, 201.1272, 185.1293, 154.9734, 112.9843	7β-Hydroxy-4(13),8-coloratadien-11,12-olide
3	27.799	C_15_H_22_O_4_	265.1445	265.1442	−1.1	265.1442, 247.1359, 229.1359, 221.1530, 219.1400, 203.1494, 185.1377, 173.8851	Ugandenial A
4	30.174	C_17_H_24_O_6_	323.1500	323.1502	0.6	323.1484, 279.1579, 263.1294, 247.1273, 219.1404, 209.1577, 189.0965, 131.0922	11*α*-Hydroxycinnamosmolide
5	31.722	C_17_H_24_O_5_	307.1551	307.1550	−0.3	307.1552, 289.1429, 274.1205, 261.1487, 247.1322, 231.1045, 219.1364, 131.3813	Ugandensolide
6	32.452	C_17_H_24_O_4_	291.1602	291.1600	−0.7	291.1600, 247.1694, 231.1363, 229.1582, 219.1796, 203.1426	Cinnamolide-3β-acetate
7	32.748	C_15_H_19_O_4_	263.1278	263.1287	3.4	263.1281, 247.1345, 235.1341, 217.1229, 191.1386, 165.8804, 118.6273	7-Hydroxywinterin
8	34.284	C_17_H_24_O_6_	323.1500	323.1498	−0.6	323.1500, 279.1575, 263.1279, 235.1313, 219.1387, 201.1291, 191.1431, 163.1467	Isomer of 4
9	35.477	C_18_H_25_O_7_	353.1595	353.1600	1.4	353.1641, 307.1552, 289.1450, 247.1322, 219.1364, 179.1321, 114.0198	Unknown
10	36.483	C_17_H_24_O_5_	307.1551	307.1550	−0.3	307.1539, 289.1450, 274.1193, 261.1477, 247.1338, 231.1019, 218.1268, 201.1297	Isomer of 5

Unknown, not identified; Rt, retention time.

## Data Availability

The data presented in this study are available are included in the article or supplementary file.

## Supplementary Materials

The following are available online at https://www.mdpi.com/article/10.3390/antiox10101559/s1, Figure S1: Cytotoxicity of WU extracts towards A549 cell lines, Figure S2: Apoptosis assays in A549 cells, Table S1: Cytotoxicity of partial plant extracts against NSCLC cell lines.
