# Supplementary material for: Stimulation of ROS Generation by Extract of Warburgia ugandensis Leading to G0/G1 Cell Cycle Arrest and Antiproliferation in A549 Cells
Source: Antioxidants (Basel). 2021 Sep 30;10(10):1559. doi: 10.3390/antiox10101559 (PMC8533466; doi:10.3390/antiox10101559)
Supplement: Supplementary file 1 [file antioxidants-10-01559-s001.zip › Table S1.pdf]

Table S1 Cytotoxicity of partial plant extracts against NSCLC cell lines

Plant	Part used	Extract solvent	Cell	IC50 (μg/mL)	Incubation time (h)	method	Referenc (PMID)
Fritillariae cirrhosae	Bulbs	aqueous	A549	37.62	48	CCK8	31669666
Elephantopus mollis	whole plant	ethyl acetate	A549	18.66 ± 5.11	48	MTT	32763415
Melissa officinalis	leaves	ethanolic	H460	100.9 ± 16.2	48	MTT	29790547
Eucalyptus globulus	aerial parts	aqueous	H460	51.7 ± 2.0	48	SRB	31165800
Green tea			A549	112	24	MTT	19137550
Toona sinensis	leaves	aqueous	H441	200	48	MTT	20478545
Toona sinensis	leaves	aqueous	H661	120	48	MTT	20478545
Toona sinensis	leaves	aqueous	H520	250	48	MTT	20478545
Bupleurum scrozonerifolium	roots	acetone	A549	60	24	MTT	15863267
Foeniculum vulgare	seeds	ethanol	H661	500-1000	48	MTS	33395604
Marsdenia tenacissima	stem	petroleum ether	A549	560 ± 50	24	CCK8	31926315
Marsdenia tenacissima	stem	ethyl acetate	A549	850 ± 40	24	CCK8	31926315
Marsdenia tenacissima	stem	n-butanol	A549	560 ± 30	24	CCK8	31926315
Selaginella doederleinii		ethyl acetate	A549	87.17 ± 0.04	48	MTT	27292193
Taxus chinensis		aqueous	H820	100-150	48	CCK8	33740524
Taxus chinensis		aqueous	A549	100-150	48	CCK8	33740524
Scutellaria baicalensis	roots	ethanol	H1299	50-100	72	MTT	34068421
Scutellaria baicalensis	roots	ethanol	A549	50-100	72	MTT	34068421
Rhus verniciflua		aqueous	A549	500-1000	24	CCK8	27667098
Alnus nitida	leaves	methanol	A549	14.91 ± 0.17	48	MTT	30508737
Alnus nitida	stem barks	methanol	A549	22.67 ± 0.17	48	MTT	30508737
Alnus nitida	leaves	methanol	H460	10.67 ± 0.12	48	MTT	30508737
Alnus nitida	stem barks	methanol	H460	25.47 ± 0.13	48	MTT	30508737
Punica granatum	leaves	ethanol	A549	50	72	MTT	27133061
Punica granatum	leaves	ethanol	H1299	47	72	MTT	27133061
