# Supplementary figures and images for: Stimulation of ROS Generation by Extract of Warburgia ugandensis Leading to G0/G1 Cell Cycle Arrest and Antiproliferation in A549 Cells
Source: Antioxidants (Basel). 2021 Sep 30;10(10):1559. doi: 10.3390/antiox10101559 (PMC8533466; doi:10.3390/antiox10101559)

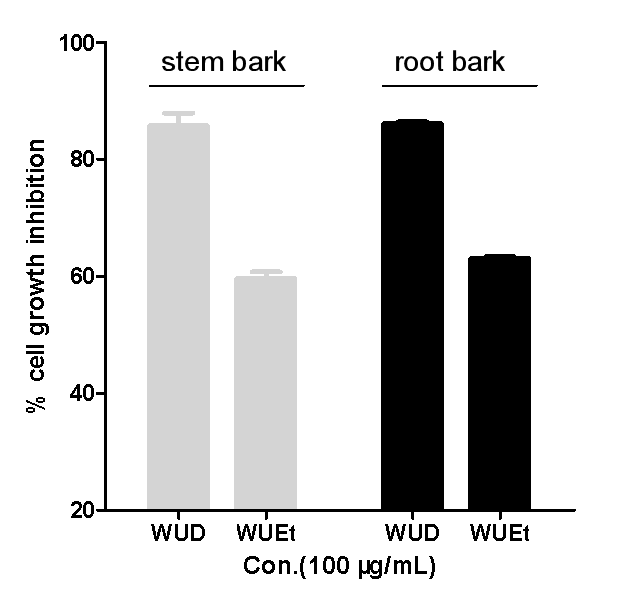

Supplement: Supplementary file 1 [file antioxidants-10-01559-s001.zip › Supplementary File-Figure S1.tif]

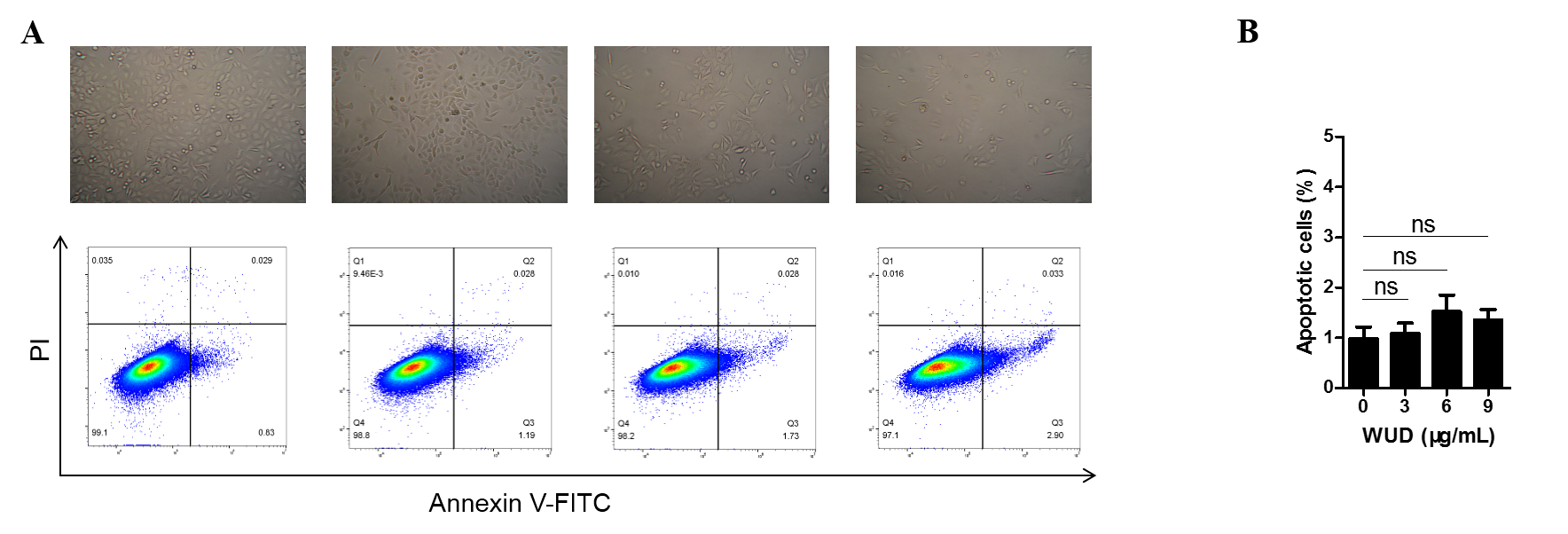

Supplement: Supplementary file 1 [file antioxidants-10-01559-s001.zip › Supplementary File-Figure S2.tif]
